# **Strengthening biopolymer adhesives through ureolysis-induced calcium carbonate precipitation**

**DOI:** 10.1038/s41598-024-84087-8

**Published:** 2025-01-27

**Authors:** Sobia Anjum, Kendall Parks, Kaylin Clark, Albert Parker, Chelsea M. Heveran, Robin Gerlach

**Affiliations:** 1 Department of Chemical & Biological Engineering, Montana State University, Bozeman, USA; 2 Center for Biofilm Engineering, Montana State University, Bozeman, USA; 3 Department of Mechanical Engineering, Montana State University, Bozeman, USA; 4 Department of Mathematical Sciences, Montana State University, Bozeman, USA; 5 Thermal Biology Institute, Montana State University, Bozeman, USA

**Keywords:** Biomineralization, Ureolysis, Biopolymer materials, Biopolymer-mineral composites, Organic-mineral, Natural adhesives, Water-based adhesives, Environmental biotechnology, Bioinspired materials, Biomineralization

## Abstract

Common adhesives for nonstructural applications are manufactured using petrochemicals and synthetic solvents. These adhesives are associated with environmental and health concerns because of their release of volatile organic compounds (VOCs). Biopolymer adhesives are an attractive alternative because of lower VOC emissions, but their strength is often insufficient. Existing mineral fillers can improve the strength of biopolymer adhesives but require the use of crosslinkers that lower process sustainability. This work introduces a novel approach to strengthen biopolymer adhesives through calcium carbonate biomineralization, which avoids the need for crosslinkers. Biomineral fillers produced by either microbially or enzymatically induced calcium carbonate precipitation (MICP and EICP, respectively) were precipitated within guar gum and soy protein biopolymers. Both, MICP and EICP, increased the strength of the biopolymer adhesives. The strength was further improved by optimizing the concentrations of bacteria, urease enzyme, and calcium. The highest strengths achieved were on par with current commercially available nonstructural adhesives. This study demonstrates the feasibility of using calcium carbonate biomineralization to improve the properties of biopolymer adhesives, which increases their potential viability as more sustainable adhesives.

**Supplementary Information:**

The online version contains supplementary material available at 10.1038/s41598-024-84087-8.

## Introduction

Most of the 7.8 million pounds of adhesives and sealants used in the United States each year are derived from petrochemical feedstocks^1^. Adhesives containing petrochemical and synthetic solvents release volatile organic compounds (VOCs), which can lead to indoor accumulation of VOCs^2,3^ and human health concerns such as sick building syndrome^4^. Studies conducted in South Korea^5^, China^6^, Iran^7^, Canada^3^, Europe, and the US^2,8,9^ show that indoor VOC exposure can have serious detrimental human health effects. Biopolymer adhesives have the potential to reduce VOC emissions^10,11^ but their adhesive strengths are limited^10,12,13^. The purpose of this study was to strengthen biopolymer (guar gum and soy protein) adhesives by using in situ, ureolysis-induced biomineral precipitation.

Biopolymer adhesives have the potential to fulfill the safety and sustainability criteria for green construction^14^ due to their renewability as well as low VOC and greenhouse gas emissions during production and use^10^. Existing biopolymer adhesives include plant-based biopolymers such as polysaccharides, proteins, oils, and tannins^10^. Among polysaccharides, natural gum adhesives have been of high interest in the food and packaging industry due to their low toxicity, availability, and film-forming properties^15^. Protein-based adhesives commonly use soy flour or soy protein because of their low toxicity, availability, and low cost^12^. However, without further modifications, biopolymer adhesives containing either polysaccharides or proteins have low adhesive shear strength compared to commercial petrochemical adhesives^12,16,17^.

The strength of biopolymer adhesives can often be increased through physicochemical treatments. These include heat- or chemical-based denaturation of proteins, enzymatic treatments, and crosslinking of the biopolymers^18–20^. Crosslinking of biopolymers stands out as the most effective method of improving strength of both polysaccharide and protein-based biopolymers, but the most commonly used crosslinkers are toxic and pose significant environmental risks^20–22^. Adhesive strength can also be improved by using mineral fillers^23–25^. Common fillers used to strengthen biopolymer adhesives have included calcium carbonate^26^, montmorillonite^27–29^, clays^27,30,31^, and nanocrystalline cellulose^32,33^. However, the addition of these fillers oftentimes requires modifications to improve the polymer-mineral interfacial bonding^34,35^. The modifications have included amidation, oxidation, polyphenolic and catechol-based crosslinking chemistries^30,31,36–41^, which increase the process complexity and cost of applications. Additional processing steps with plasticizers and crosslinkers are still required to optimize the rheological behavior of the nanocomposite adhesives^26,31^. The additional processing necessary to produce desirable adhesive properties increases cost and decreases sustainability. Therefore, it is desirable to develop synthesis strategies for mineral-reinforced biopolymer adhesives that simplify processes and reduce the potential for negative impacts on sustainability.

Biomineral fillers produced by ureolysis could strengthen biopolymer adhesives in a manner that involves straightforward processing steps and has the potential for improved sustainability. In ureolysis, urea is hydrolyzed by urease to ammonium and carbonate ions, which precipitate out as calcium carbonate in the presence of sufficient calcium ions. This process is referred to as ureolytically induced calcium carbonate precipitation (UICP) and can be summarized as follows^42^:

1 $$ \:{\text{H}}_{2} {\text{N}} - {\text{CO}} - {\text{NH}}_{2} \:\left( {{\text{urea}}} \right)\: + \:2{\text{H}}_{2} {\text{O}}\: + \:{\text{Ca}}^{{2 + }} \xrightarrow{{{\text{urease}}}}\:2{\text{NH}}_{4}^{ + } \: + \:{\text{CaCO}}_{3} \left( {\text{s}} \right) $$

Ureolysis can be carried out at ambient temperatures by ureolytic bacteria^42^ or fungi^43^ as well as solutions or suspensions of the urease enzyme derived from microbial or vegetable sources^44^. The biomineralization induced by UICP, including both microbial and enzymatic sources, has been frequently used to bind or coarsen particles in applications such as soil stabilization, concrete remediation, well leakage remediation, and the creation of subsurface barriers^42,45^. UICP can also be conducted in situ within hydrogels, to produce strong and tough biomineralized materials^46–49^. These results suggest that favorable interactions can be developed between UICP-precipitated biominerals and surrounding polymer matrices. Therefore, UICP may be an effective strategy to produce biomineral fillers that succeed in strengthening biopolymer adhesives, sustainably and cost effectively^50,51^.

The goal of this study was to determine the influence of in situ precipitation of biomineral fillers on the adhesive shear strength of biopolymer adhesives produced by microbially induced calcium carbonate precipitation (MICP) or enzymatically induced calcium carbonate precipitation (EICP), on the adhesive shear strength of biopolymer adhesives. In this investigation, the impacts of MICP and EICP on adhesive strength were studied for two types of biopolymers. Guar gum and soy protein were selected as representatives of polysaccharide- and protein-based adhesives, respectively. Guar gum is a versatile polymer, commonly used as a viscosity enhancer and stabilizer in the food industry^52^, and soy protein is a promising biopolymer matrix used in emerging biobased adhesives^20^. Furthermore, additional optimization with respect to cell density and biomineralization solution chemistry was performed to increase the strength of the adhesives. These results demonstrate considerable strengthening (up to 6 times stronger) of adhesives through UICP-induced biomineral fillers on common surfaces (i.e., glass and stainless-steel).

## Results and discussion

### Adhesive performance of biopolymers is improved by ureolytically induced calcium carbonate precipitation

**Fig. 1:**
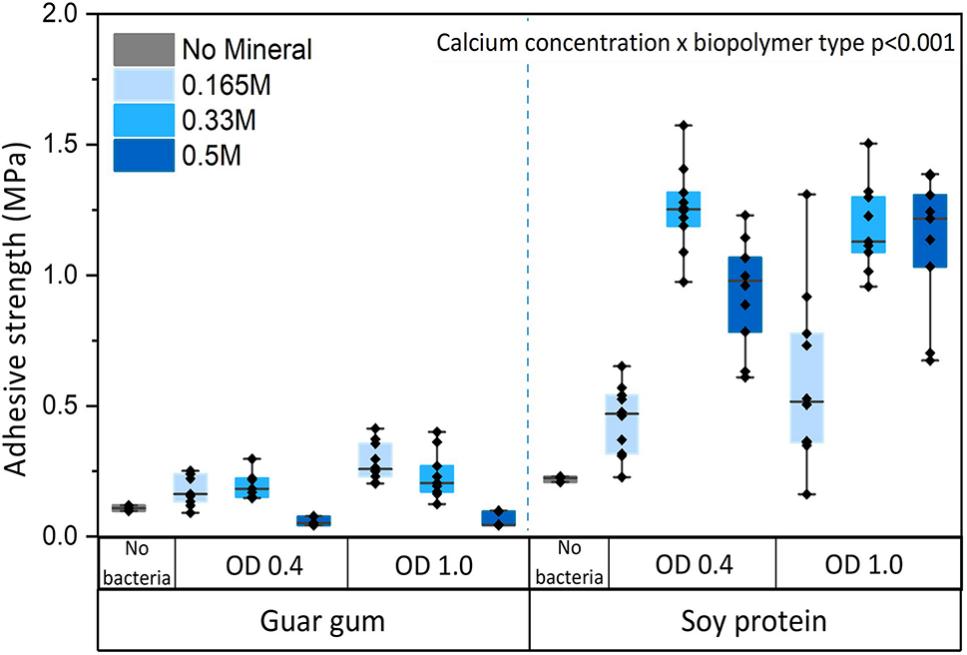
Adhesive shear strength of guar gum- and soy protein-based microbially induced calcium carbonate adhesives on glass lapjoints. The calcium concentrations and bacterial cell density (OD) were varied for each biopolymer (guar gum and soy protein) to optimize the adhesive shear strengths of MICP-reinforced adhesives. Control samples (“No bacteria”) do not contain bacteria and only either soy protein or guar gum in DI water. The upper and lower bounds of the boxplot represent the 25th and 75th percentiles, and the whiskers indicate minimum and maximum values. The median is indicated by a horizontal straight line.

Biopolymer matrices were successfully mineralized with calcium carbonate using UICP, either MICP or EICP. Field-Emission Scanning Electron Microscopy and confocal laser scanning microscopy show that the mineral was incorporated and distributed throughout the biopolymer matrix (Supplementary Figs. S1, S2 and S3). X-ray diffraction and thermogravimetric analyses show that the mineral phase formed is predominantly calcium carbonate (Supplementary Figs. S2 and S4). This work demonstrates that UICP reinforcement, utilizing MICP or EICP, can increase the adhesive strength of biopolymer adhesives by ~ 3 to 6 times. The primary determinant of adhesive strength as well as strength gain in response to UICP-reinforcement was the type of biopolymer matrix (i.e., soy protein or guar gum) (Fig. 1).

**Fig. 2:**
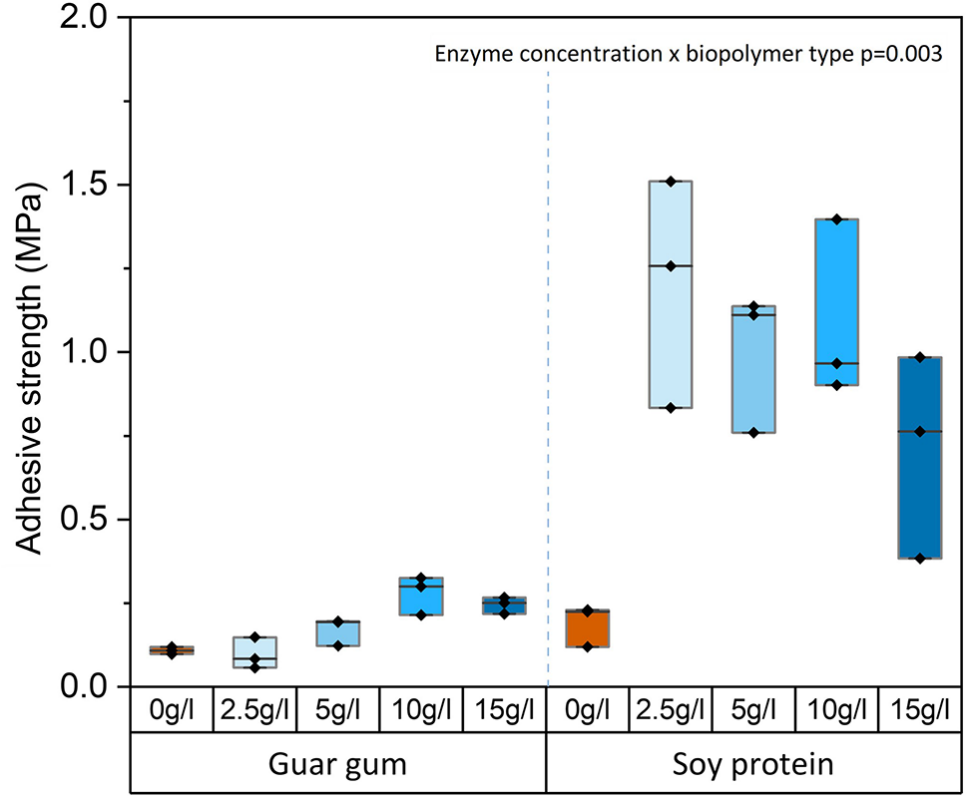
Adhesive shear strength of EICP-reinforced biopolymer adhesives on glass lapjoints. For each biopolymer, urease enzyme concentrations equivalent to 2.5–15 g/l of Jack Bean Meal were tested at 0.165 M calcium concentrations. Control samples (‘0 g/l’) contain only soy protein or guar gum in DI water. The upper and lower bounds of the boxplot represent the 25th and 75th percentiles, and the whiskers indicate minimum and maximum values. The median is indicated by a horizontal straight line.

The baseline adhesive strength for soy protein was higher than for guar gum (0.22 ± 0.01 MPa for soy protein vs. 0.11 ± 0.01 MPa for guar gum on glass). While soy protein adhesives successfully bonded glass and stainless-steel, guar gum adhesives were only successful on glass (Supplementary Table S1). After biomineralization, the strength of soy protein adhesives improved by ~ 6 times (up to 1.26 ± 0.2 MPa with MICP and 1.24 ± 0.3 MPa with EICP, Figs. 1 and 2). In contrast, the strength of guar gum biopolymer adhesives on glass increased by ~ 3 times (up to 0.28 ± 0.07 MPa with MICP and 0.28 ± 0.04 MPa with EICP). Therefore, the type of biopolymer matrix is important in determining both adhesive strength as well as the types of surfaces where the adhesives can be successfully used. Notably, for several MICP soy protein adhesives, the glass slides failed before the adhesive (Supplementary Table S1). This substrate failure shows that these results may underestimate the adhesive strength of some of the stronger MICP soy protein adhesive preparations.

The improvements to biopolymer adhesive strength with UICP reinforcement aligns with the strength gains seen from abiotic or geologic minerals from other studies^26,27,29^. However, the biomineralization method introduced here does not require any crosslinking of the biopolymer or functionalization of the mineral surfaces to achieve the improvement in strength. Therefore, the use of UICP for in situ biomineralization of biopolymer adhesives achieves the strength benefits of these prior methodologies while reducing the number of processing steps and improving sustainability (i.e., no use of solvents that produce VOCs).

**Fig. 3:**
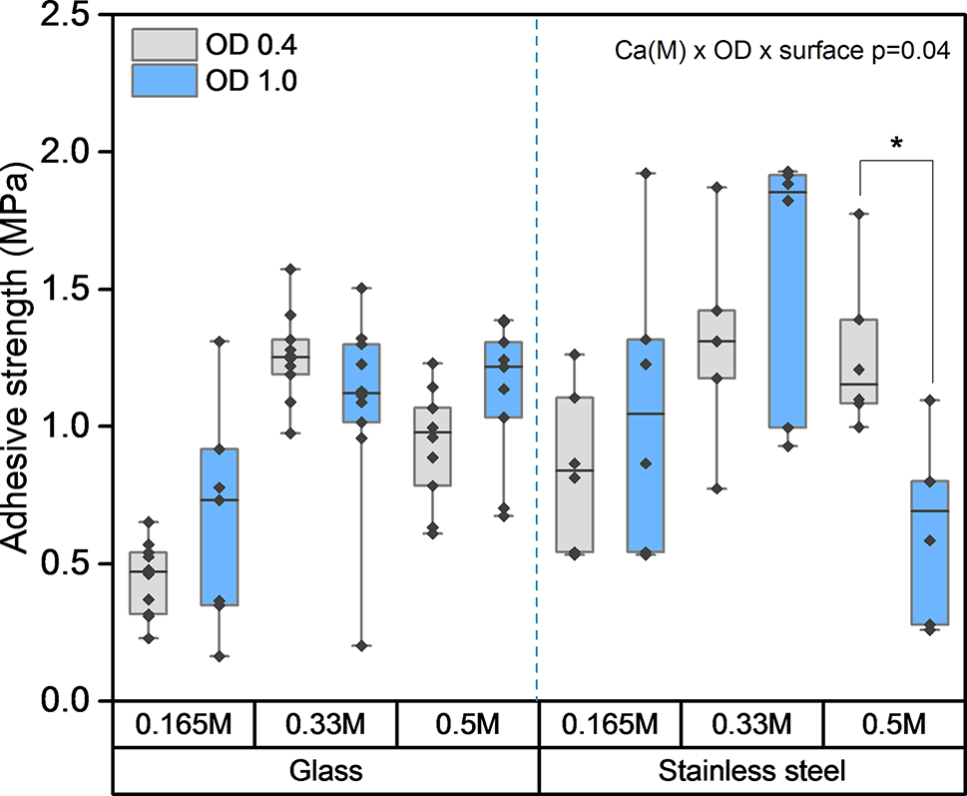
The adhesive shear strength of MICP-reinforced soy protein adhesives on stainless-steel and glass substrates for a calcium concentration range from 0.165 M to 0.5 M and bacterial cell densities (OD) of 0.4 and 1.0. The upper and lower bounds of the boxplot represent the 25th and 75th percentiles, and the whiskers indicate minimum and maximum values. The median is indicated by a horizontal straight line.

Both MICP and EICP showed similar potential to increase the adhesive strength of biopolymers. These strengths, as high as 1–2 MPa (Figs. 1, 2 and 3), are comparable with strengths of commercially available rubber^53^ and acrylic adhesives^54^ and are suitable for non-structural adhesive applications (e.g., bonding floor and wall coverings, panels, fibers, particle boards, etc.). Since their strengths are similar, choosing MICP or EICP for biomineralization of biopolymer adhesives may depend on competing practical considerations. While MICP requires live microbial cultures, which require longer preparation times (24 h or more), EICP can be performed relatively easily and quickly (minutes to a few hours) using powdered plant ureases. On the other hand, plant-based urease sources with high urease activity (i.e., jack bean meal) cost more than the raw materials needed for microbial growth^55^ and require using agricultural land for non-food purposes. In a time-cost trade-off, MICP would be more suitable for large construction projects, while EICP might be preferable for smaller custom applications.

### Calcium concentration influences the adhesive shear strength of MICP-reinforced biopolymer adhesives

MICP-reinforcement of biopolymer adhesives could potentially be optimized through tuning parameters that determine the kinetics and amount of mineral precipitation, including the bacterial cell and calcium concentrations^56,57^. Therefore, these parameters were optimized for both guar gum- and soy protein-based adhesives. The calcium concentration in solution controls the total calcium carbonate precipitation in the adhesives. From the stoichiometry of the ureolytic reaction (Eq. 1), it is expected that for a calcium concentration increase from 0.165 M to 0.5 M, the mineral content of the biopolymer adhesives would increase from 1.65 to 5.0% w/w (Supplementary Table S2). However, adhesive strength was not only related to the calcium content in the biomineralization solution but instead showed an interaction between biopolymer type and calcium concentration, (*p* < 0.001, Supplementary Table S3). Post-hoc testing reveals that the highest adhesive strengths were achieved for mid-range calcium concentrations for soy protein isolate and for the lowest calcium concentration for guar gum (1.26 and 0.28 MPa, achieved at 0.33 M and 0.165 M calcium concentrations, respectively, Fig. 1). An increase in the calcium concentrations to 0.5 M resulted in a significant decrease in the adhesive strength for both guar gum and soy protein adhesives.

These data agree with the literature, which indicates that the shear and tensile strength of mineral-reinforced polymers does not linearly increase with an increase in mineral content but instead flatlines or declines beyond an optimum value^58,59^. Prior literature suggests that as mineral quantity increases, strength increases but the ductility of the polymer matrix decreases^58,60,61^. When brittleness is increased as an effect of crosslinking, stress concentrations can increase at the edges of bonded regions in lap shear mode, resulting in premature joint failure^62^.

The cellular biomass can also be an important potential control parameter for UICP-reinforced biopolymer adhesives^63,64^. However, our data show that varying the bacterial cell concentration (between an OD of 0.4 and an OD of 1.0) did not influence strength gain significantly (*p* = 0.33, Supplementary Table S3). It has been reported that very high biomass concentrations can shift the kinetic dependency of ureolysis from enzyme-limited to substrate-limited^65^. Hence, for the cell density and urea concentrations used in this study, the ureolytic reaction appears to be substrate-limited.

Beyond the mineral content, the strength of UICP-reinforced biopolymer adhesives is likely to depend on mineral dispersion^59^ and mineral-microbe-biopolymer interactions^66–68^. Biopolymers can attach to surfaces through physiochemical attractions and physical interlinking of polymeric chains^10^. Guar gum is a polysaccharide with an abundance of hydrophilic moieties while soy protein displays both hydrophobic and hydrophilic regions, contributing to hydrogen bonding and Van der Waals interactions suitable for adhesion to a variety of surfaces^20,52^. The addition of mineral fillers enhances the adhesive strength of biopolymers^60,69,70^. One potential reason for the increased strength is enhanced interfacial bonding between mineral and the polymer^71,72^. In biomineralization the candidate mechanisms for interfacial interactions include physical entanglement of biopolymer in the biomineral during its growth and adhesive electrostatic interactions at the biomineral-biopolymer interfaces^67,73^. These interactions depend on biopolymer types and physiochemical parameters of mineral formation^73^. Investigating which of these candidate mechanisms individually or together confer the strengthening during biomineralization of guar gum and soy protein biopolymers would benefit from future investigation.

### Enzyme concentrations and biopolymer type influence the adhesive shear strength of EICP-reinforced biopolymer adhesives

The strength of EICP-reinforced biopolymer adhesives could potentially depend on jack bean urease and calcium concentrations; therefore these factors were investigated. The highest adhesive strengths were found for a calcium concentration of 0.165 M for EICP-reinforced guar gum biopolymers (Supplementary Fig. S5). This calcium concentration was then selected for studies of the influence of urease concentration on shear strength. The highest adhesive strengths achieved for EICP-reinforced guar gum and soy protein were 0.33 and 1.51 MPa, achieved at 10 g/l and 2.5 g/l jack bean urease concentrations, respectively (Fig. 2, Supplementary Table S5).

The impact of urease concentration on strength depended on the biopolymer type (interaction: *p* < 0.001, Supplementary Table S6). Increasing the urease concentration from 2.5 to 10 g/l significantly increased the adhesive shear strength of EICP-reinforced guar gum adhesives (+ 65%, *p* < 0.001) but did not significantly affect the adhesive shear strength of EICP-reinforced soy protein adhesives (*p* = 0.71, Fig. 2). A further increase in urease concentration from 10 g/l to 15 g/l significantly decreased the adhesive shear strength of EICP soy protein adhesives (*p* < 0.001). Therefore, it appears that there is a mid-range of urease concentration that maximizes adhesive strength.

MICP and EICP showed differences in the relationship between enzyme concentration and strength gain. Hence, the amount of urease available in, both, the MICP and EICP preparations was compared. Assuming a urease content of 0.1% for JBM and 1% for *S. pasteurii* biomass^74,75^ and estimating the biomass concentration for *S. pasteurii* (dry weight vs. OD), the urease content was compared for MICP and EICP. The biomass concentration for *S. pasteurii* was estimated to be 1.1 g/L (dry weight) for the OD 1.0 treatments and 0.44 g/L for the OD 0.4 treatments, respectively (data not shown). The urease content was comparable for the 5 g/L JBM treatment in EICP, and the 0.4 OD treatments in MICP (0.005 g/L urease vs. 0.0044 g/L urease for EICP and MICP, respectively). The urease content for the 10 g/L JBM and the 1.0 OD treatments were also comparable (0.01 g/L urease vs. 0.011 g/L urease for EICP and MICP, respectively). Nevertheless, the MICP process appeared to be substrate-limited; the strength gain depended on urea and calcium concentrations but not on enzyme concentrations (which increases with higher bacterial cell density). By contrast, the EICP process appeared to be enzyme-limited because higher strengths were achieved with higher urease availability (up to 10 g/L JBM).

There are several reasons that may contribute to these differences. Firstly, the specific activity and efficiency of ureolysis by *S. pasteurii* and jack bean urease can differ based on pH, temperatures, solution chemistry, bacterial growth conditions and enzyme purity^74,76^. For instance, the filtered JBM solutions for EICP were produced in deionized water while *S. pasteurii* cell suspensions were added in fresh nutrient broth. Secondly, bacterial cells can actively attach to substrata, such as glass or stainless-steel while enzyme sorption is a passive process. Thirdly, the MICP and EICP fundamentally differ in enzyme-substrate interactions. In EICP, interactions between urea and urease depend on the diffusion and passive transport of urea to and ammonium & carbonate ions away from the enzyme. By contrast, during MICP the bacterial cells can actively control the flux of urea into and carbonate & ammonium ions out of the bacterial cells^63,77^. Overall, these differences in enzyme activity, efficiency and availability potentially influence the enzyme and substrate limited nature of ureolysis in MICP and EICP.

### The type of surface influences the adhesive shear strength of MICP-reinforced biopolymer adhesives

The adhesive strength of the MICP-reinforced adhesives was compared on glass and stainless-steel. These materials are both non-porous but have different composition, surface wettability and roughness (Supplementary Fig. S6). It was found that guar gum or MICP-reinforced guar gum did not bond stainless-steel lapjoints (Supplementary Table S1). However, the MICP-reinforced soy protein had comparable and at times even higher adhesive strength on stainless-steel than on glass surfaces (Fig. 3).

For MICP soy protein adhesives, there was a three-way interaction between calcium concentration, cell density, and surface type on adhesive shear strength (*p* = 0.04, Supplementary Table S7). For both glass and stainless-steel surfaces, the mid-range calcium concentration (0.33 M) best promoted adhesive strength (Fig. 3). The adhesive strength was lower for a bacterial cell concentration equivalent to an OD of 1 compared to an OD of 0.4, but only for 0.5 M calcium concentration and on stainless-steel surfaces. In other comparisons, cell density did not influence strength.

As discussed previously, the tendency of the glass to break before the adhesive failed for some of the MICP-reinforced soy protein replicates (0.33 M and 0.5 M) indicates that the true adhesive strength for these MICP-reinforced soy protein adhesives is underestimated. Together, these results demonstrate that MICP-reinforced adhesives develop desirable strength characteristics on at least two common surfaces (glass and stainless-steel) with different surface characteristics (for example, surface roughness and wettability).

### Failure characteristics of MICP-reinforced adhesives depend on the surface type

**Fig. 4:**
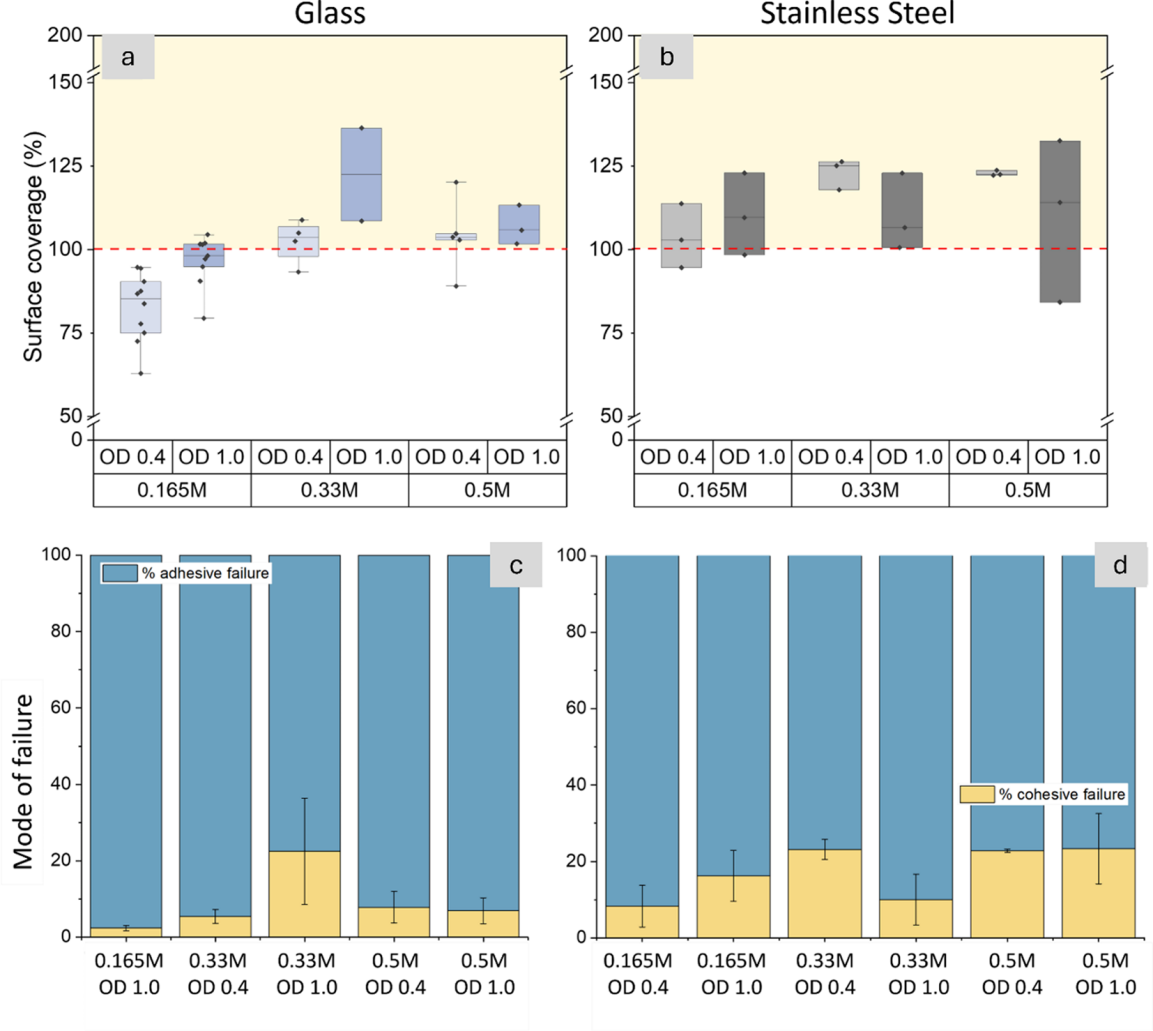
Surface coverage and failure mode analysis for MICP-reinforced soy protein adhesives with calcium concentration range 0.165 M to 0.5 M and bacterial cell densities (OD) of 0.4 and 1.0. Summative surface coverage of adhesive residue was measured after the failure of (**a**) glass and (**b**) stainless-steel lapjoints. The values on the y-axis indicate the surface coverage summed for both sides of the bonded regions. Thus, 100% surface coverage indicates complete adhesive failure while 200% indicates complete cohesive failure; values between 100 and 200 indicate mixed failure [yellow shaded regions in (**a**) and (**b**)]. The upper and lower bounds of the boxplot represent the 25th and 75th percentiles, and the whiskers indicate minimum and maximum values. A horizontal straight line indicates the median. (**c**,** d**) Further evaluation of the data indicates that adhesive failure (blue) was predominant over cohesive failure (tan) across all samples. Stacked bars indicate the sample means, and whiskers indicate 1 standard error of the mean.

To add further insight into how MICP reinforcement improves adhesive properties, surface coverage and mode of failure were compared across different surfaces and calcium concentrations. In the following analysis, 100% total summative surface coverage indicates complete adhesive failure, while 200% indicates complete cohesive failure (“Methods” section, Eqs. 2–4). For most of the tested lapjoints, the summative surface coverage was between 100 and 200%, indicating that a mixed failure (both adhesive and cohesive failure) occurred (Fig. 4a,b, Supplementary Table S8). The percentages of adhesive and cohesive behavior in the mixed failure were estimated by Eqs. (3), (4) and are shown in Fig. 4c,d. Values less than 100% indicate that some material is lost during testing. The treatment at a calcium concentration of 0.165 M and an OD of 0.4 was excluded from the mode of failure analysis because significant material was lost during testing, resulting in a summative surface coverage of less than 100% (Fig. 4a).

Our data demonstrate that failure behaviors of the bonded regions were affected both by the calcium concentration and surface type (Fig. 4, Supplementary Table S9). Overall, the MICP-reinforced adhesives mostly failed adhesively (Fig. 4c and d), but stainless-steel surfaces had a significantly higher fraction of cohesive failure than glass (*p* = 0.002, Supplementary Table S9). On stainless-steel, increasing calcium concentrations from 0.165 M to 0.5 M improved the cohesive behavior of the mixed failure but did not consistently improve strength (Figs. 3 and 4d). On glass, this same increase in calcium concentration improved strength but did not change the extent of cohesive failure (Figs. 3 and 4c). These data indicate that increasing calcium concentrations can improve adhesion at the adhesive-adherend interface, but the gain in strength is not explained by the extent of cohesive behavior (Supplementary Fig. S7). These results agree with the literature that suggests the relationship between strength and mode of failure is variable and multifactorial^71,78,79^. Strength and cohesive behavior can likely both be improved by surface preparation^78^, which would benefit from future investigation.

The increased tendency for cohesive failure on stainless-steel, relative to glass, can be attributed to the higher surface roughness and hydrophobicity of stainless-steel surfaces^12^. Surface profilometry showed that the surface roughness of stainless-steel (S_a_=0.45 ± 0.0008 μm) was an order of magnitude greater than for glass (S_a_=0.046 ± 0.0023 μm, Supplementary Fig. S6). The surface wettability analysis showed that stainless-steel surfaces also had a higher hydrophobicity than glass (Supplementary Fig. S6). Studies have shown that higher surface roughness and greater hydrophobicity can each improve microbial adhesion^80,81^, biopolymer-surface interactions^82^, and calcium carbonate deposition^83^. A combination of these factors may be responsible for improved adhesion of the MICP-reinforced biopolymer adhesives on stainless-steel. Further investigation of mineral-biopolymer-surface interactions are needed for understanding the mechanisms of strength development in UICP-reinforced adhesives.

### Sustainability of UICP-reinforced biopolymer adhesives and prospects

Our results demonstrate that reinforcing biopolymer adhesives using MICP or EICP (both forms of ureolysis-induced calcium carbonate precipitation (UICP)) improves their strength sufficiently to be useful for indoor applications. These UICP-reinforced adhesives have the potential for improved sustainability in terms of the renewability of reactants and the lower environmental and human health effects of their by-products. A principal reason for this improved sustainability is that no organic solvents are used during the production of these adhesives and, therefore, they avoid VOC emissions. Importantly, ureolysis generates ammonium ions, which in environments with pH > 9 can convert to ammonia, a volatile inorganic compound with a noxious smell and known health effects^84^. However, ammonium ions themselves do not cause these issues. In the manner that these UICP-reinforced adhesives are synthesized, the pH is maintained below 8, and almost all ammonium stays in solution. Further, ammonium forms ammonium chloride in the chemical environment of these adhesives, as confirmed by X-ray diffraction analysis (Supplementary Fig. S2). At higher temperatures (> 300 °C), the ammonium chloride decomposes into ammonia and HCl gas, requiring ventilation measures to be in place. During indoor use at ambient temperatures, the risk of ammonia release from UICP adhesives is minimal. Further assessments of the fate of ammonium ions can provide additional validation of the environmental applicability of the UICP-reinforced biopolymer adhesives.

## Conclusions

This work demonstrates that reinforcement of common biopolymers through ureolysis-induced calcium carbonate precipitation (UICP) can produce adhesives with strength on par with commercial adhesives for nonstructural indoor applications. Both microbial and enzymatic sources of the urease enzyme successfully strengthened the adhesives without the need for additional processing (e.g., thermal treatments or chemical crosslinkers). Aside from biomineralization, the most critical factor determining the strength of the biomineral reinforced biopolymer adhesives was the type of biopolymer used. Specifically, at the biopolymer concentrations used, soy protein outperformed guar gum in adhesive strength. These types of adhesives have the potential for increased sustainability because of the renewability of reactants, zero VOC emissions, and low environmental and human health concerns of their by-products. The promising strength and sustainability characteristics of these adhesives motivate future studies focused on identifying and optimizing strengthening mechanisms of biopolymer-biomineral interactions as well as the adhesive durability characteristics.

## Materials and methods

### Materials

Jack bean powder, Brain Heart Infusion, and guar gum were purchased from Sigma Aldrich. Soy protein isolate was purchased from MP Biomedicals. BD Difco™ Nutrient broth, urea (purity ≥ 99%), calcium chloride dihydrate (≥ 97%), and ammonium chloride (≥ 99.5%) were purchased from Fisher Scientific. All solutions were prepared in deionized water with an electrical resistivity of ≥ 18.2 MΩ.

### Microbially induced calcium carbonate precipitation (MICP)

Microbially induced calcium carbonate precipitates were prepared by mixing a culture of the bacterium *Sporosarcina pasteurii* with a mineralization solution. Bacterial cultures were prepared by inoculating 100 ml of filter-sterilized growth medium (0.33 M urea, 20 g/l Brain Heart Infusion broth) with 1 ml of a thawed *S. pasteurii* (ATCC11859) stock culture. This starter culture was incubated overnight at 30 °C and 150 rpm on a horizontal shaker before 1 ml of the overnight culture was transferred into 100 ml of fresh growth medium (0.33 M urea, 0.18 M NH_4_Cl, 3 g/l Nutrient broth) and incubated again overnight at 30 °C at 150 rpm. This bacterial culture was harvested at 20–22 h after achieving an optical density of 0.4 ± 0.05 (mean ± SD) measured at 600 nm (OD_600_). The bacterial cultures at OD 1.0 were prepared by centrifuging the overnight culture at 3000×g for 5 min at 21 °C. The cell pellet was harvested and resuspended in sterile growth medium to reach an OD of 1.0 ± 0.05. The optical densities were measured in a flat bottom 96-well plate (Polystyrene, Greiner Bio-One) using a Synergy HT Spectrophotometer (Biotek); at 200 µl of liquid, the path length was estimated to be 5.7 mm. Absorbance values for the sterile growth medium were subtracted from sample readings to remove the contribution of the growth medium and the plate itself. The mineralization solution had the same composition as the growth medium but contained different concentrations of additional urea and calcium chloride (0.33 M, 0.66 M, or 1 M in equimolar ratios). The bacterial cultures and mineralization solutions were mixed in a 1:1 ratio to achieve calcium concentrations of 0.165 M, 0.33 M, and 0.5 M.

Guar gum or soy protein were added to the formulations at 0.7% (w/v) and 10% (w/v), respectively, to produce MICP-reinforced guar gum and soy protein adhesive suspensions, respectively. These concentrations were chosen to provide sufficient viscosity to apply the adhesive to lapjoints as well as sufficient adhesive strength for the lapjoints without mineral reinforcement.

### Enzymatically induced calcium carbonate precipitation (EICP)

Jack bean meal (JBM) solution was prepared by suspending 5, 10, 20, and 30 g/l of fine powdered JBM in deionized water for 4 h. Before use, the resulting suspensions were filtered through 0.22 μm pore size bottle top filters (Nalgene Rapid-Flow Filters, Thermo Scientific). Mineralization solutions were prepared with urea and calcium chloride at 0.33 M, 0.66 M, and 1 M concentrations. JBM and mineralization solutions were mixed in a 1:1 ratio to achieve 0.165 M, 0.33 M, and 0.5 M urea and calcium concentrations. Guar gum and soy protein were added at 0.7% (w/v) and 10% (w/v) to make EICP-reinforced guar gum and soy protein adhesive suspensions, respectively.

### Lapjoint assembly and adhesive shear strength testing

**Fig. 5:**
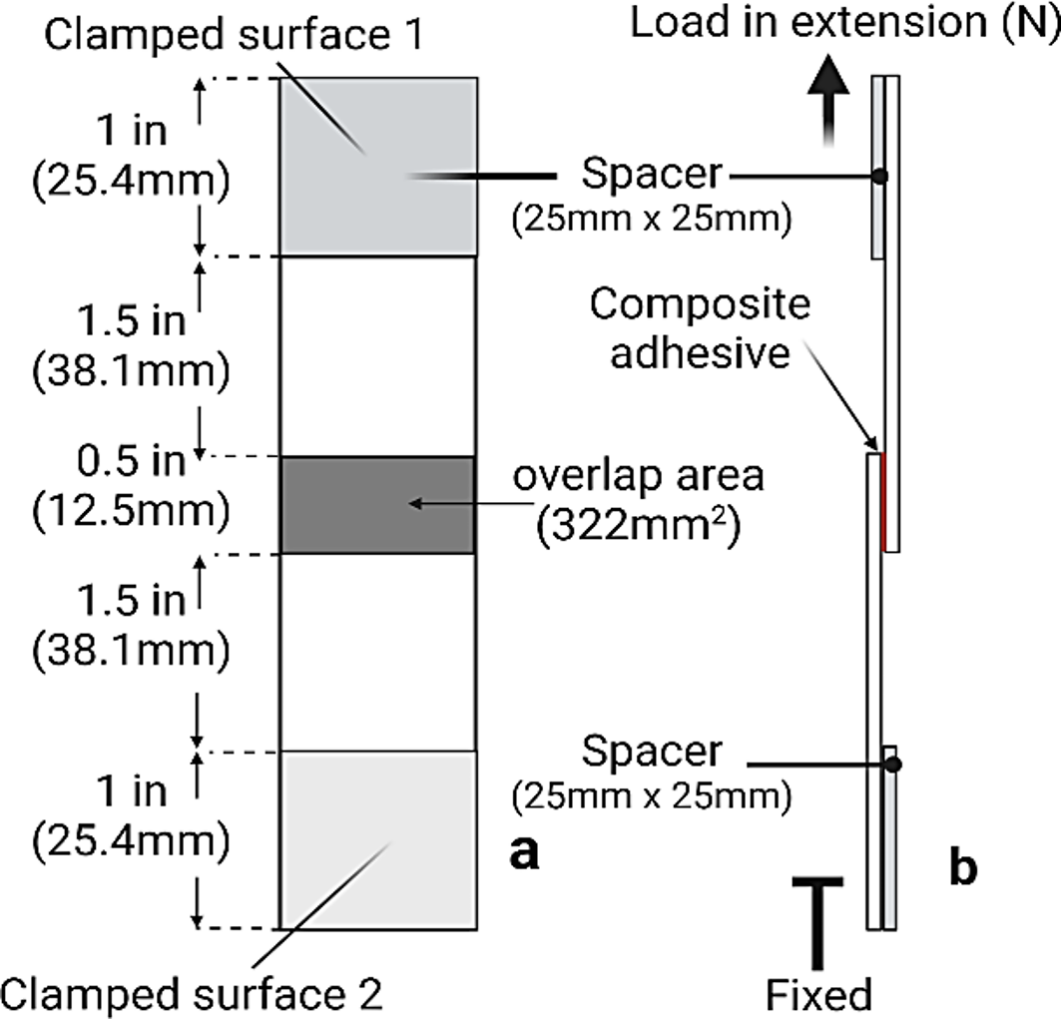
Lapjoint dimensions and testing configuration. (**a**) Lapjoints were prepared by applying adhesive to the marked overlap area, cured, and then tested by (**b**) lap shear in tensile extension mode.

Stainless-steel (316 L) and borosilicate glass adherends (25 mm × 75 mm) were cleaned with deionized water and 70% ethanol. Square spacers (25 mm × 25 mm) were attached to each adherend (Fig. 5). The adhesive solution was vortexed for 5 s, and 100 µl of the composite was applied to a 12.5 mm × 25 mm edge of one adherend placed flat on a bench top. The other adherend (i.e., a second slide) was placed on top to create a 12.5 mm × 25 mm overlap area (Fig. 5, ASTM method D1002-10). No additional external pressure was applied. The sample was allowed to cure for 48 h at 23 ± 2 °C.

After curing, the samples were tested at a tensile extension rate of 0.01 mm/sec in ramp-to-fail mode using an Instron 3340 Series Universal Testing System. The maximum load (N) at the point of bond failure divided by the overlap area (322 mm^2^) was used to calculate the adhesive shear strength of the sample in megapascals (N/mm^2^).

### Surface roughness and surface wettability

Surface roughness (S_a_) of glass and stainless-steel surfaces was estimated using a Filmetrics Profilm 3D optical profilometer at 50× magnification on triplicate (300 μm × 300 μm) regions. The surface wettability of adherends was estimated by measuring the contact angle of deionized water on the glass and stainless-steel using a video contact angle system (VCA2500XE, AST).

### Surface coverage and mode of failure analysis

The failure mode of the bonded regions was calculated based on three predicted modes of failure: adhesive, cohesive, and mixed (adhesive and cohesive). In a complete cohesive failure, both lapjoint sides would have a near 100% surface coverage, and the summative surface coverage would be 200%. In a complete adhesive failure, the summative surface coverage would be 100%, indicating full coverage on one side and no coverage on the opposing side. The values between 100 and 200% represent mixed failure and can be used to estimate the extent of adhesive and cohesive behavior using Eqs. (3) and (4). Both sides of fractured lapjoints were imaged post-fracture (iPhone 12) and thresholded in ImageJ using Otsu’s method^85^. The surface coverage on each side (assigned a and b) of the fractured lapjoint was calculated using the ImageJ analyze particle function to measure areas covered by the adhesive (Supplementary Fig. S8). The summative coverage of the adhesive in the bonded regions was calculated by Eq. (2). Since most of the studied lapjoints showed a mixture of cohesive and adhesive failure, the extent of cohesive and adhesive behavior was further evaluated by Eqs. (3)–(4).

2 $$\:\text{S}\text{u}\text{m}\text{m}\text{a}\text{t}\text{i}\text{v}\text{e}\:\text{s}\text{u}\text{r}\text{f}\text{a}\text{c}\text{e}\:\text{c}\text{o}\text{v}\text{e}\text{r}\text{a}\text{g}\text{e}\:\left(\text{S}\text{S}\text{C}\right)=(\text{a}\text{d}\text{h}\text{e}\text{s}\text{i}\text{v}\text{e}\:\text{s}\text{u}\text{r}\text{f}\text{a}\text{c}\text{e}\:\text{c}\text{o}\text{v}\text{e}\text{r}\text{a}\text{g}\text{e}\:\text{o}\text{n}\:\text{s}\text{i}\text{d}\text{e}\:\text{a}+\text{s}\text{i}\text{d}\text{e}\:\text{b})$$

3 $$\:\text{C}\text{o}\text{h}\text{e}\text{s}\text{i}\text{v}\text{e}\:\text{f}\text{a}\text{i}\text{l}\text{u}\text{r}\text{e}\:\left(\text{C}\text{F}\right)=(\text{S}\text{S}\text{C}-100)$$

4 $$\:\text{A}\text{d}\text{h}\text{e}\text{s}\text{i}\text{v}\text{e}\:\text{f}\text{a}\text{i}\text{l}\text{u}\text{r}\text{e}=(100-\text{C}\text{F})$$

### Statistical analysis

For MICP-reinforced adhesives, three-way ANOVAs assessed whether adhesive strength depended on biopolymer type (soy protein or guar gum), calcium concentration, bacterial OD, or the interactions between these factors. Additional three way-ANOVAs also tested whether adhesive strength and percentage of adhesive failure depended on surface type (stainless-steel or glass), calcium concentration, bacterial OD, or their interactions. For EICP-reinforced adhesives, a two-way ANOVA tested whether adhesive strength depended on biopolymer type, enzyme concentration, or their interactions. Critical alpha was set as 0.05 a priori. Residuals were checked for normality and equal variance for all models. Post-hoc testing was performed for significant interactions using a Tukey correction to adjust critical alpha for family-wise error.

## Electronic supplementary material

Below is the link to the electronic supplementary material.

Supplementary Material 1

## Data Availability

The datasets used and/or analyzed during the current study are available from the corresponding author on reasonable request.
