# Supplementary material for: Strengthening biopolymer adhesives through ureolysis-induced calcium carbonate precipitation
Source: Sci Rep. 2025 Jan 27;15:3453. doi: 10.1038/s41598-024-84087-8 (PMC11772823; doi:10.1038/s41598-024-84087-8)
Supplement: Supplementary file 1 — Supplementary Material 1 [file 41598_2024_84087_MOESM1_ESM.pdf]

Supplementary Information

Strengthening biopolymer adhesives through ureolysis-induced calcium carbonate precipitation

Sobia Anjum[a,b], *Kendall Parks*[a,b], *Kaylin Clark*[b,c], *Albert Parker*[b,d], *Chelsea M. Heveran*[b,c], *and Robin Gerlach*[*a,b,e]

[a]Department of Chemical & Biological Engineering, Montana State University, USA

[b]Center for Biofilm Engineering, Montana State University, USA

[c]Department of Mechanical Engineering, Montana State University, USA

[d]Department of Mathematical Sciences, Montana State University, USA

[e]Thermal Biology Institute, Montana State University, USA

[*]Corresponding Author, T +1 406-994-1840, Email: robin_g@montana.edu

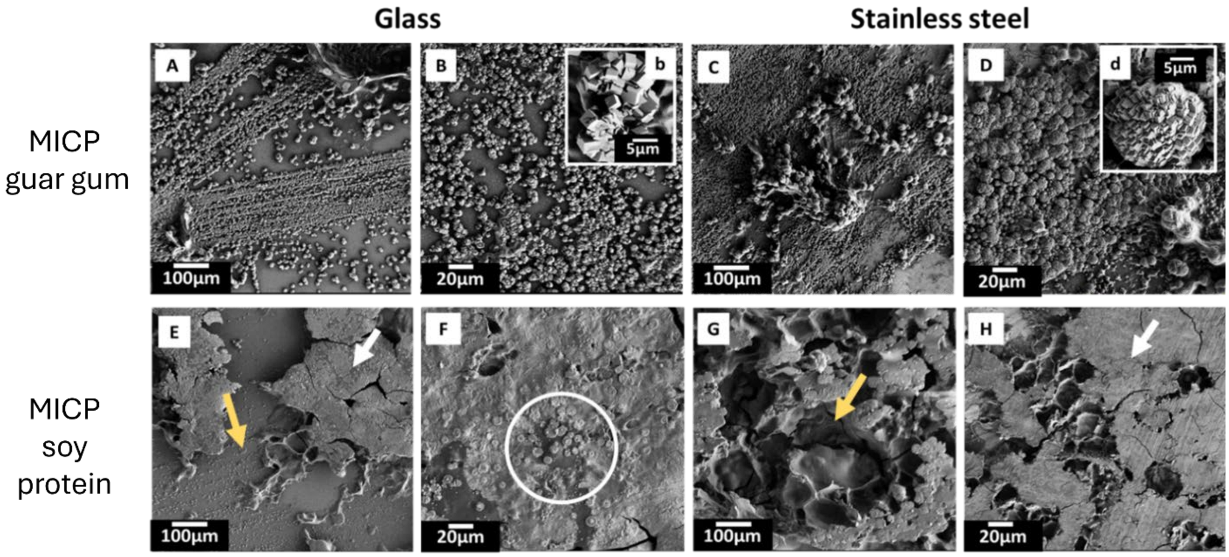

Figure S1. Field Emission Scanning Electron Microscope (FESEM) images of MICP-reinforced guar gum and MICP-reinforced soy protein adhesives post-fracture. Discrete spherical aggregates in MICP guar gum on (A, B) glass and (C, D) stainless steel. Inset images b & d show that the aggregates are polycrystalline with sharp edges and smooth crystal faces, indicative of polycrystalline calcite. Biopolymer-mineral aggregates in MICP-soy protein on (E, F) glass and (G, H) stainless steel surfaces (EDX in Figure S2). (E) A yellow arrow indicates the glass surface in the background, and a flat region indicated by the white arrow shows potential sites of adhesive failure (delamination) of MICP soy protein on glass surfaces. (F) The MICP soy protein adhesives have circular aggregates (potentially mineral precipitates) embedded in the (soy protein) matrix, encircled white. The site of (G) fracture within the adhesive is indicated by the yellow arrow, and (H) potential delamination of adhesive is shown by a white arrow.

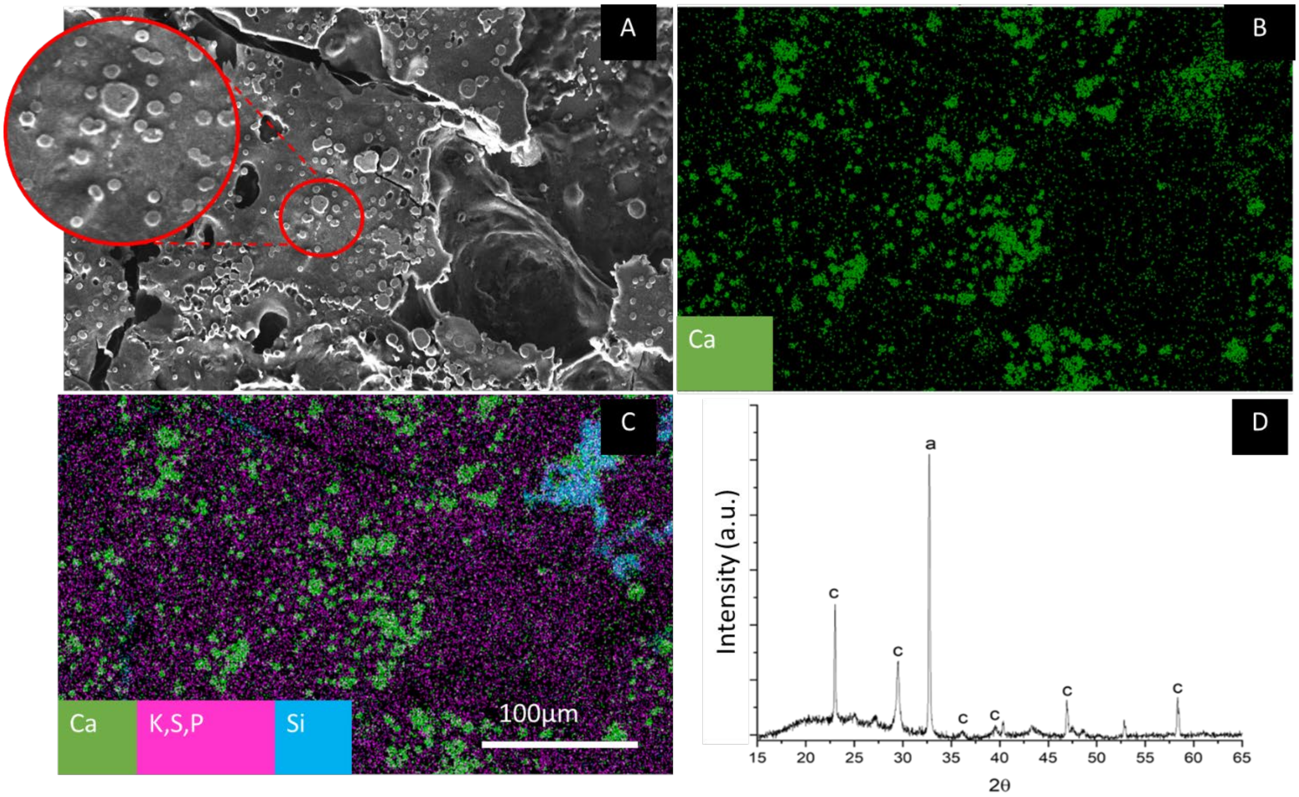

Figure S2. Field Emission Scanning Electron Microscopy Backscattered Electron (FESEM-BSE) imaging and Energy Dispersive Spectroscopy (EDS) elemental mapping of MICP-reinforced soy protein adhesive on a lapjoint post fracture. (A) The FESEM-BSE image of a MICP-reinforced soy protein adhesive shows distinct, relatively circular aggregates (encircled red) within the mineral-biopolymer adhesive. FESEM-EDS shows that these are calcium-rich regions. (C) The presence of K, S, and P indicates the presence of an organic (biopolymer) matrix. (D) The presence of calcium carbonate precipitates in the adhesives was confirmed using XRD, indicating a predominance of calcite polymorph (indicated by peaks labeled 'c'). The peak labeled 'a' indicates the presence of precipitated ammonium chloride in the adhesive.

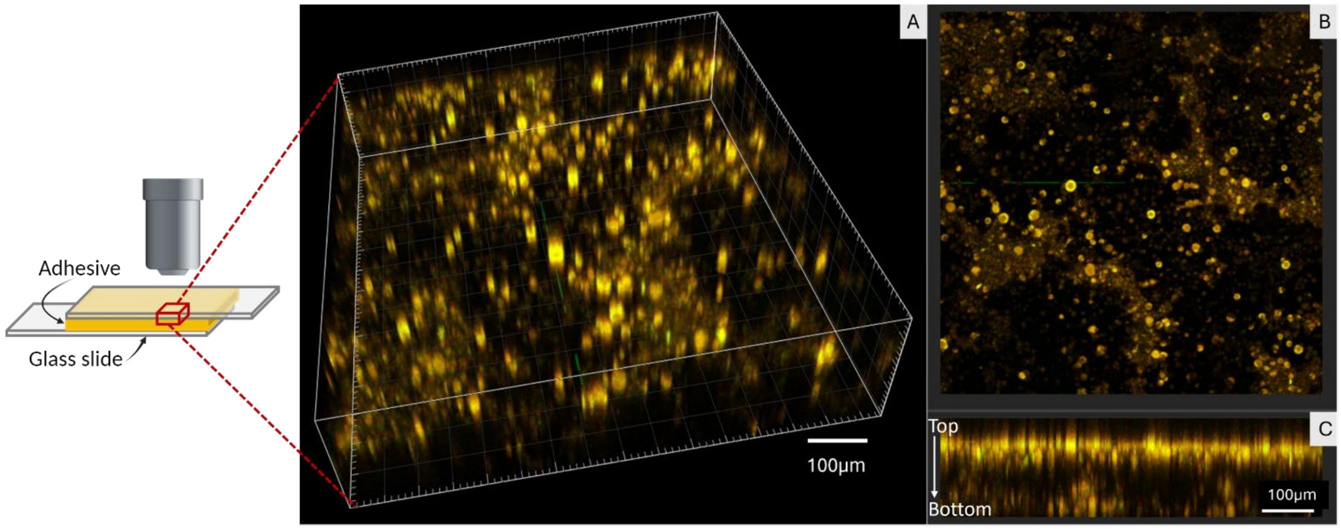

Figure S3. Confocal laser scanning microscopy 3D reconstruction of a MICP-reinforced guar gum adhesive on a glass lapjoint after fracturing. Previous research demonstrated that microbially induced calcium carbonate exhibits autofluorescence at 405 nm excitation and an emission range of 415 nm to 550 nm [1]. (A) Three-dimensionally distributed calcium carbonate biominerals were observed after excitation of the sample at a wavelength of 405nm, the emission signal was captured between 500 and 700nm wavelengths. The black background is the adhesive sample that does not fluoresce in this excitation-emission range. (B) The Top-down and (C) side view of the image volume show that minerals are distributed throughout the adhesive. The bottom of the image volume receives lower exposure to excitation wavelengths due to the opacity of the calcium carbonate precipitates. This results in a potential imaging artifact indicating a higher mineral density at the top than at the bottom of the image volume.

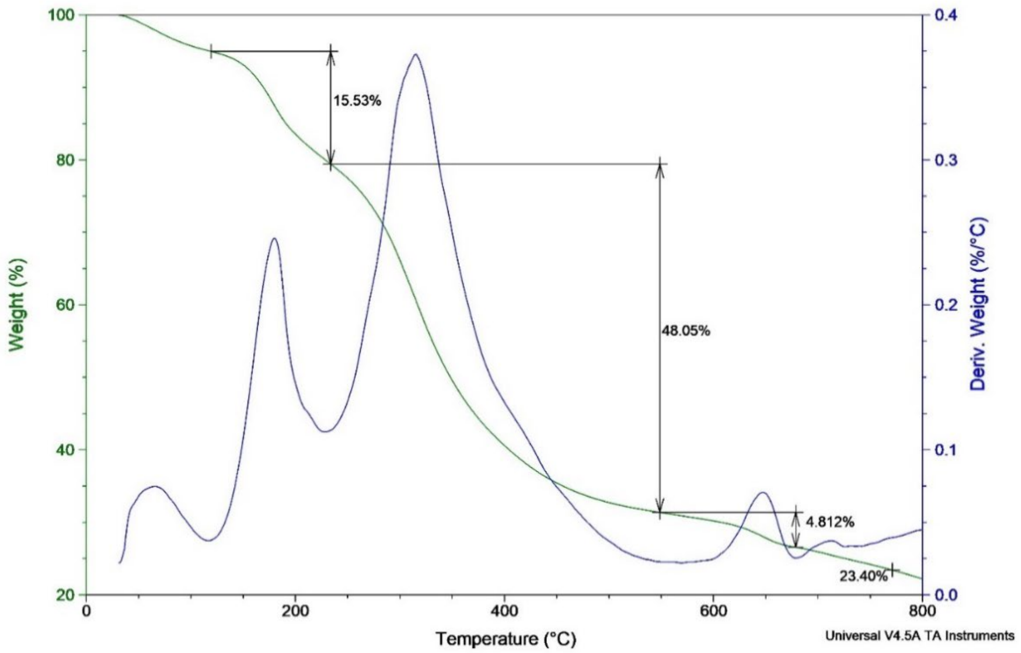

Figure S4. The thermogravimetric profiles of the MICP-reinforced soy protein adhesive were developed by tracking weight loss (normalized to initial weight, w/w_i, weight (%)) and rate of weight loss ($\Delta(w/w_i)/\Delta T$, Deriv. Weight (%/˚C)) from 50 to 800˚C, at a heating rate of 10˚C/min. The weight loss from 50 to 100˚C is associated with the loss of water. The volatilization and degradation of low molecular weight organics is expected between 100 to 200˚C, soy protein and ammonium chloride between 200 to 550˚C, and calcium carbonate between 600 to 750˚C. The calcium carbonate content was measured from percent weight loss between 600 to 750˚C, assuming that 4.81% of weight loss was associated with the release of CO_2 during calcium carbonate degradation. Based on the mass balance ($CaCO_3$ (s) → CaO (s) + CO_2 (g)), it was estimated that 10.93% of the weight was $CaCO_3$ in the MICP-reinforced soy protein formulation with 0.165M calcium concentration, which is close to the maximum theoretical value of 12.72 % (Table S2).

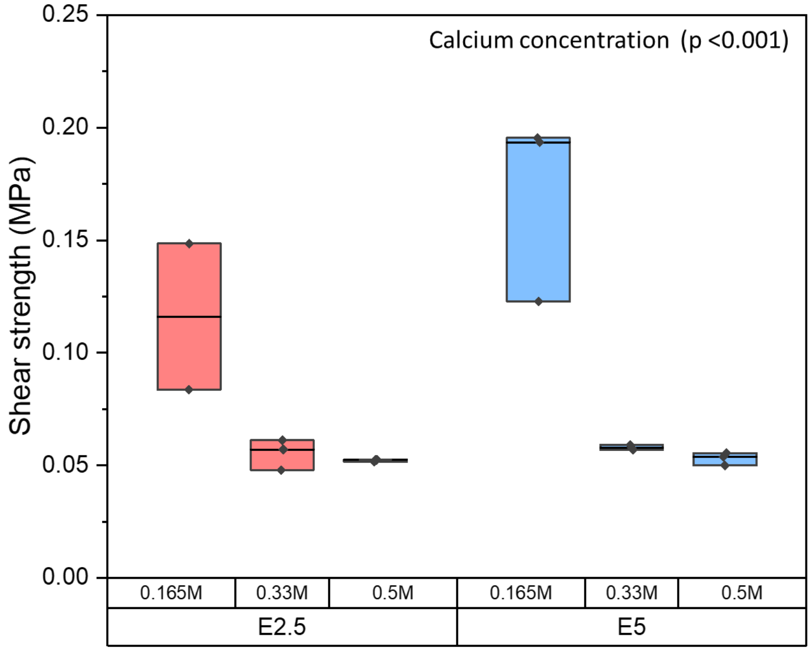

Figure S5. Shear strength of EICP guar gum at varying urea and calcium concentrations (M), and enzyme (E) concentrations 2.5 (red) and 5g/l (blue). The upper and lower bounds of the boxplot represent the 25th and 75th percentiles, and the whiskers indicate the minimum and maximum values. The median is indicated by a horizontal straight line. Statistical analysis in Table S4.

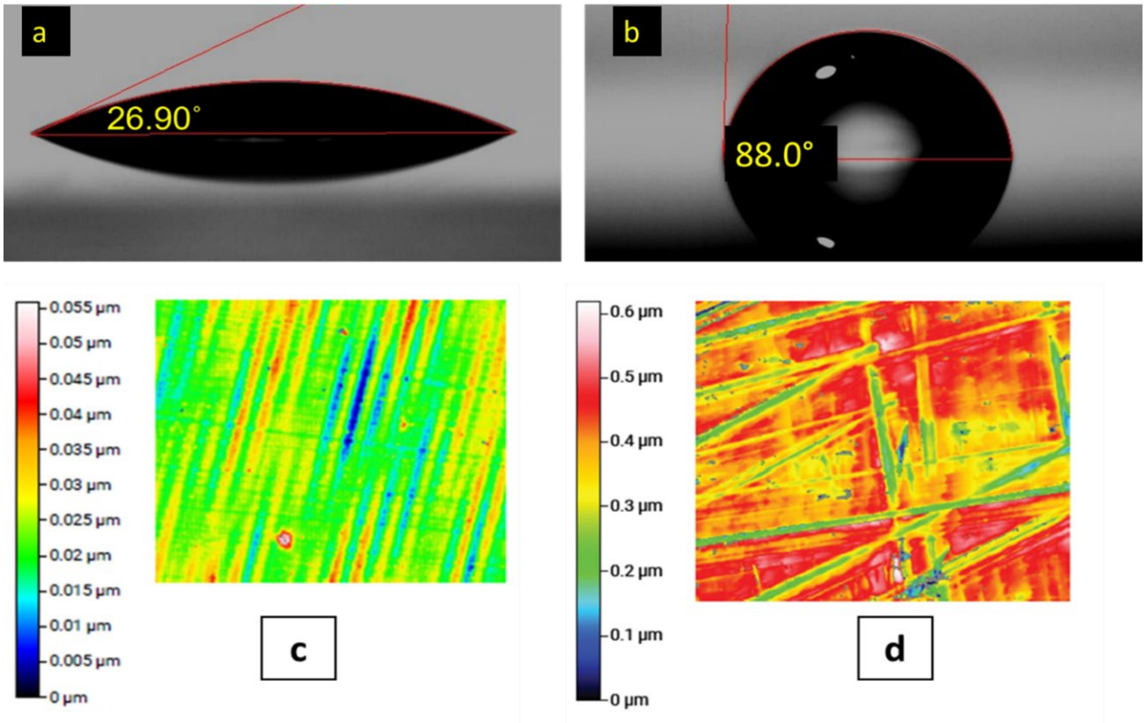

Figure S6. Hydrophobicity and roughness of glass and stainless-steel surfaces were measured using a Video Contact Angle System and a Profilm 3D optical profilometer, respectively. The contact angle of water (yellow text) on (a) glass is 26.9˚, and on (b) stainless steel is 88˚, indicating that the stainless-steel surface is slightly hydrophobic, and the glass surface is highly hydrophilic. Surface roughness of (c) glass and (d) stainless steel surface, color legends show an increasing surface roughness with a transition from blue to red. (c,d) Color maps of surface roughness show that the glass surface has a lower surface roughness (N=3, 0.046 ± 0.0023µm), than the stainless steel surface (N=3, S_a 0.45 ± 0.0008µm).

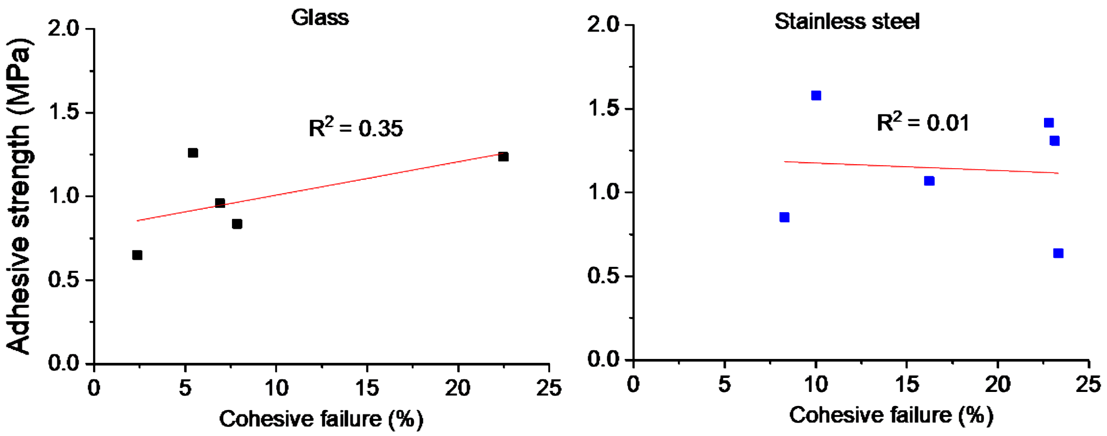

Figure S7. A weak correlation was observed between shear strength and cohesive failure for glass ($R^2 = 0.35$) and none for stainless steel surfaces ($R^2 = 0.01$).

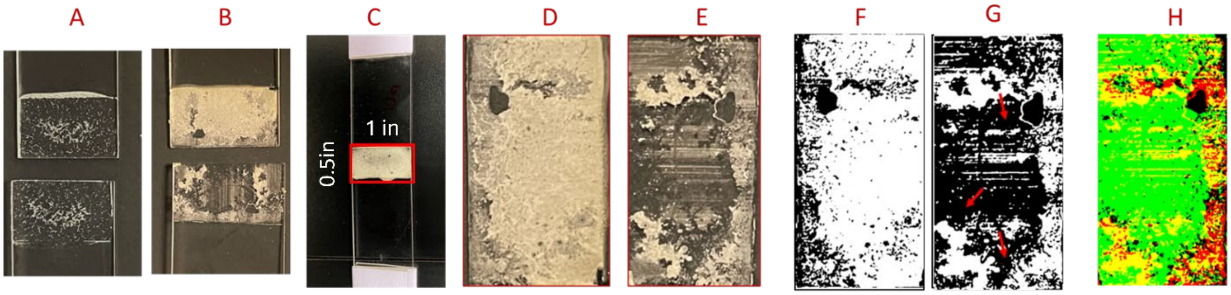

Figure S8. Failure analysis of glass lapjoints post fracture. (A) MICP guar gum adhesives had incomplete surface coverage of the bonded region (0.5 in x 1.0 in) within the lapjoints. (B) MICP soy protein adhesives show mixed failure (i.e., both adhesive and cohesive characteristics), and (C) achieve complete coverage of the bonded region (inside the red rectangle) area between glass surfaces. (D, E) Surface coverage of the adhesive on each side of the lapjoint after failure were measured by thresholding the images using the Otsu method, where (F&G) white indicates the biopolymer adhesive coverage against black adherend background and red arrows indicate locations of adhesive failure. (H) Regions in yellow show areas of cohesive failure, where the adhesive remains on both sides of the lapjoint. In this image, red and green indicate the coverage of adhesive on either side of the lapjoint side, black indicates the absence of adhesive.

Table S1. The adhesive strength of microbially induced calcium carbonate (MICP) adhesives on glass and stainless-steel lap joints. The table shows calcium concentrations and microbial cell density tested for each biopolymer type. n = number of specimens tested to failure.

Adhesive type		Biopolymer	Calcium (M) +	*S. pasteurii* OD600	Adhesive strength Mean ±SD (MPa)	
					Glass	Stainless steel
1	MICP - soy protein	Soy protein isolate (100g/l)	0.165	0.4	0.45 ±0.1 (n=9) [a]	0.85 ±0.3 (n=6)
2				1.0	0.65 ±0.3 (n=9) [a]	1.1 ±0.6 (n=6)
3			0.33	0.4	1.26 ±0.2 (n=5) [a]	1.3 ±0.4 (n=6)
4				1.0	1.24 ±0.2 (n=4) [a]	1.6 ±0.5 (n=6)
5			0.5	0.4	0.84 ±0.2 (n=6) [a]	1.26 ±0.3 (n=6)
6				1.0	0.96 ±0.3 (n=4) [a]	0.64 ±0.3 (n=6)
7	Soy protein – no MICP		DI water		0.22 ±0.01 (n=6)	0.25 ±0.1 (n=6)
8	MICP - guar gum	Guar gum (7g/l)	0.165	0.4	0.17 ±0.06 (n=8) [b]	-
9				1.0	0.28 ±0.07 (n=10) [b]	-
10			0.33	0.4	0.20 ±0.05 (n=7) [b]	-
11				1.0	0.24 ±0.09 (n=9) [b]	-
12			0.5	0.4	0.06 ±0.02 (n=5) [b]	-
13				1.0	0.06 ±0.03 (n=6) [b]	-
14	Guar gum – no MICP		DI water		0.11 ±0.01 (n=6) [b]	-

¶ [a] Soy protein reinforced replicates with MICP experienced substrate failure (*i.e.,* the glass slides broke before the adhesive bond failed). [b] Some guar gum replicates fell apart before testing; their results are not included here.

¶ - (Dashes) indicate that no data are available. Guar gum and MICP-reinforced guar gum did not provide measurable adhesive strength on stainless steel lap joints for the curing and testing parameters used in this work.

Table S2. The table shows the dry weight of components and expected content of calcium carbonate formed during ureolysis in MICP-reinforced adhesives.

MICP-reinforced soy protein adhesives			
	0.165M calcium	0.33M calcium	0.5M calcium
Nutrient broth (g/l)	3	3	3
Urea (g/l)	9.9	19.8	30
Soy protein (g/l)	100	100	100
Calcium Chloride (g/l)	18.31	36.62	55.49
Expected $CaCO_3$ content (g/l)	16.5	33	50
Expected $CaCO_3$ content (w/w) %	1.65	3.3	5.0
Total dry wt. of components (g/l)	131.21	159.42	188.49
(wt. $CaCO_3$ / total dry wt.) %	12.57*	20.7	26.52
MICP-reinforced guar gum adhesives			
	0.165M calcium	0.33M calcium	0.5M calcium
Nutrient broth (g/l)	3	3	3
Urea (g/l)	9.9	19.8	30
Guar gum (g/l)	7	7	7
Calcium Chloride (g/l)	18.31	36.62	55.49
Expected $CaCO_3$ content (g/l)	16.5	33	50
Expected $CaCO_3$ content (w/w) %	1.65	3.3	5.0
Total dry wt. (g/l)	38.21	66.42	95.49
(wt. $CaCO_3$ / total dry wt.) %	43.18	49.68	52.36

*The weight percent of calcium carbonate in MICP-reinforced soy protein (0.165M Ca) measured by thermogravimetric analysis was ~11% (85% of the expected calcium carbonate content), indicating that most of the calcium in the adhesive precipitated as calcium carbonate (*cf.* Figure S4).

Table S3. Results of a three-factor ANOVA for MICP soy protein on glass lap joints (independent variables: biopolymer, calcium concentrations (Ca), and bacterial cell density (OD)).

Analysis of Variance for response shear strength					
Source	DF	Adj SS	Adj MS	F-Value	P-Value
Ca	3	3.31	1.10	30.23	0.00
Biopolymer	1	7.78	7.78	213.54	0.00
Cell O.D.	1	0.04	0.04	0.96	0.33
Ca x Biopolymer	3	3.64	1.21	33.25	0.00
Ca x Cell OD	3	0.20	0.07	1.84	0.15
Biopolymer x Cell OD	1	0.00	0.00	0.01	0.94
Ca x Biopolymer x Cell OD	3	0.15	0.05	1.43	0.24
Error	101	3.68	0.04		
Total	116				

Table S4. Results of a two-factor ANOVA to test the effect of calcium (Ca) and urease concentration on the adhesive strength of EICP-reinforced guar gum on glass lap joints (independent variables: calcium concentration, urease concentration).

Analysis of Variance for response shear strength					
Source	DF	Adj SS	Adj MS	F-Value	P-Value
Ca	2	0.027	0.014	26.34	0.00
Urease concentration	1	0.001	0.002	3.03	0.11
Ca x Urease concentration	2	0.002	0.001	2.34	0.14
Error	11	0.005	0.000		
Total	16	0.040			

Table S5. The adhesive strength of EICP-reinforced biopolymer adhesives tested at increasing urease concentrations tested for each biopolymer type on glass lap joints.

Adhesive type		Biopolymer	Calcium (M) + Jack bean meal urease g/l		Adhesive strength Mean ±SD (MPa)
1	EICP soy protein	Soy protein isolate (100 g/l)	0.165M	2.5 (n=3)	1.2 ±0.3
2				5 (n=3)	1.1 ±0.2
3				10 (n=3)	1.0 ±0.3
4				15 (n=3)	0.71 ±0.3
5	EICP control		DI water (n=3)		0.22 ±0.01
6	EICP guar gum	Guar gum (7 g/l)	0.165M	2.5 (n=3)	0.1 ±0.025
7				5 (n=3)	0.17 ±0.06
8				10 (n=3)	0.28 ±0.04
9				15 (n=3)	0.25 ±0.05
10	EICP control		DI water (n=3)		0.11 ±0.01

Table S6. Results of a two-factor ANOVA to test the effect of biopolymer type and urease concentration on the adhesive strength of EICP-reinforced biopolymer adhesives on glass lap joints (independent variables: biopolymer, urease concentration).

Analysis of Variance for log-transformed (ln) response shear strength					
Source	DF	Adj SS	Adj MS	F-Value	P-Value
Enzyme concentration	4	5.40	1.35	16.66	0.00
Biopolymer	1	16.46	16.46	202.90	0.00
Enzyme concentration x Biopolymer	4	3.18	0.79	9.79	0.00
Error	20	1.62	0.08		
Total	29	26.66			

Table S7. Results of a three-factor ANOVA for to test the effect of surface type, calcium concentration and bacterial cell density on the adhesive strength of MICP-reinforced soy protein adhesives (independent variables: surface type, calcium concentrations (Ca), and bacterial cell density (OD)).

Analysis of Variance for log-transformed (ln) response shear strength					
Source	DF	Adj SS	Adj MS	F-Value	P-Value
Ca	2	1.14	0.57	20.56	0
Surface	1	0.19	0.19	6.85	0.01
Cell OD	1	0.00	0.00	0.02	0.88
Ca x Surface	2	0.18	0.09	3.25	0.05
Ca x Cell OD	2	0.20	0.10	3.67	0.03
Cell OD x Surface	1	0.05	0.05	1.72	0.20
Ca x Cell OD x Surface	2	0.19	0.10	3.44	0.04
Error	61	1.69	0.03		
Total	72	4.09			

Table S8. Surface coverage and failure data for MICP-soy protein adhesives on glass and stainless-steel lap joints. The table shows the surface coverage of the adhesive and microbial cell density tested for each biopolymer type.

Biopolymer type	Calcium (M)	OD	Surface coverage (a+b) Mean ±SD	Failure analysis (Cohesive failure %)
MICP- soy protein glass (tested n=10)	0.165	0.4	84.77 ±8.12 (n=9)	(n=0)*
		1.0	96.63 ±7.73 (n=9)[a]	(n=5)* 2.37 ±3.95
	0.33	0.4	102.39 ±6.62 (n=5)[a]	(n=4)* 5.43 ±4.56
		1.0	122.50 ±19.70 (n=4)[a]	(n=2)* 22.47 ±5.59
	0.5	0.4	104.10 ±11.02 (n=6)[a]	(n=5)* 7.85 ±3.95
		1.0	106.92 ±5.87 (n=4)[a]	(n=3)* 6.92 ±4.56
MICP- soy protein stainless steel (tested n=6)	0.165	0.4	103.72 ±9.64 (n=3)[b]	(n=2) 8.30 ±5.91
		1.0	110.29 ±12.29 (n=3)	(n=2)16.24 ±5.91
	0.33	0.4	123.13 ±4.57 (n=3)	(n=3) 23.13 ±4.83
		1.0	110.04 ±11.55 (n=3)	(n=3) 10.04 ±4.83
	0.5	0.4	122.82 ±0.78 (n=3)	(n=3) 22.82 ±4.83
		1.0	110.30 ±24.4 (n=3)	(n=2) 23.33 ±5.91

[a]The variability in the number of replicates used in surface coverage analysis is due to the exclusion of lap joints where the failure occurred in the substrate instead of the bonded region, reducing the number of samples available for image analysis.

*Number of samples included in failure analysis is lower than the number of samples used for surface coverage analysis; only samples with a total surface coverage (a+b) greater than 100%, which indicated cohesive failure, were included in the failure analysis. Adhesive failure was back-calculated from the estimated percentage cohesive failure.

Table S9. Results of a three-factor ANOVA for MICP soy protein (dependent variable: summative surface coverage, independent variables: surface type, calcium concentrations (Ca), and bacterial cell density (OD)).

Analysis of Variance for response summative surface coverage					
Source	DF	Adj SS	Adj MS	F-Value	P-Value
Ca	2	608.8	304.42	3.86	0.037
OD	1	110.6	110.61	1.4	0.25
Surface	1	961.9	961.95	12.19	0.002
Ca*OD	2	173.4	86.68	1.1	0.352
Ca*Surface	2	284.3	142.16	1.8	0.19
OD*Surface	1	264.8	264.78	3.36	0.081
Error	21	1656.6	78.89		
Lack-of-Fit	1	341.6	341.55	5.19	0.034

[1] N. M. Zambare, N. Y. Naser, R. Gerlach, and C. B. Chang, "Mineralogy of microbially induced calcium carbonate precipitates formed using single cell drop-based microfluidics," Sci Rep, vol. 10, no. 1, Dec. 2020, doi: 10.1038/s41598-020-73870-y.
